# Micronutrient gaps during the complementary feeding period in 6 countries in Eastern and Southern Africa: a Comprehensive Nutrient Gap Assessment

**DOI:** 10.1093/nutrit/nuaa142

**Published:** 2021-03-08

**Authors:** Jessica M White, Ty Beal, Joanne E Arsenault, Harriet Okronipa, Guy-Marino Hinnouho, Kudakwashe Chimanya, Joan Matji, Aashima Garg

**Affiliations:** 1 United Nations Children's Fund (UNICEF), New York, New York, USA; 2 Global Alliance for Improved Nutrition, Washington, DC, USA; 3 Department of Environmental Science and Policy, University of California, Davis, Davis, California, USA; 4 Institute for Global Nutrition, University of California, Davis, Davis, California, USA; 5 Intake, Center for Dietary Assessment, FHI Solutions, Washington, DC, USA; 6 Department of Population Medicine and Diagnostic Sciences, Cornell University, Ithaca, New York, USA; 7 Helen Keller International, New York, New York, USA; 8 UNICEF, Regional Office for Eastern and Southern Africa, Nairobi, Kenya

**Keywords:** adequacy, assessment, dietary intake, micronutrient deficiencies, nutrient gap

## Abstract

Insufficient quantity and inadequate quality of foods in early life are key causes of all forms of malnutrition. Identification of nutrient and dietary gaps in the diets of infants and young children is essential to inform policies and programs designed to improve child diets. A Comprehensive Nutrient Gap Assessment was used to assess the public health significance of nutrient gaps during the complementary feeding period and to identify evidence gaps in 6 countries in Eastern and Southern Africa. Important gaps were identified in iron, vitamin A, zinc, and calcium and, to a lesser extent, vitamin B_12_ and folate. The best whole-food sources of these micronutrients available in part or all of the countries studied include beef liver, chicken liver, small dried fish, beef, and eggs. Investment is needed in many countries to collect data on micronutrient biomarkers and dietary intake. Strategic actions to improve child diets will require engagement and intervention across relevant systems to accelerate progress on improving the diets of infants and young children.

## INTRODUCTION

Approximately 1 in 5 of the 144 million children younger than 5 years worldwide who have stunted growth reside in the UNICEF Eastern and Southern Africa region. In 2019, 33% of children younger than 5 years in the region had stunted growth and 6% were wasted.^1^ Only 2 of the 12 countries with available data in the region are on track to achieve the 2025 global nutrition target of reducing by 40% the number of children affected by stunting.^2^ Hidden hunger also persists in the region. For example, although prevalence of vitamin A deficiency (VAD) in children younger than 5 years in low- and middle-income countries declined from 39% to 29% between 1991 and 2013, no progress was achieved in sub-Saharan Africa: VAD prevalence was 45% in 1991 and is 48% today.^3^ An estimated 69% and 64% of children in that age group suffer from either vitamin A or iron deficiency in Eastern Africa and Southern Africa, respectively.^4^

Insufficient quantity and inadequate quality of foods during the complementary feeding period—between 6 and 23 months of age—are key causes of all forms of malnutrition, including micronutrient deficiencies, and have immediate and long-term consequences. In the short term, these include increased morbidity and mortality and delayed cognitive and motor development.^5^ In later childhood, adolescence, and adulthood, poor nutrition in early life can result in impaired academic and work capacity, reproductive outcomes and overall health, hindering economic development and contributing to the intergenerational cycle of malnutrition.^5–7^ Yet, in Eastern and Southern Africa, child diets remain suboptimal: Only 24% of children 6–23 months of age are fed the minimum number of recommended food groups per day and 43% are fed the minimum recommended number of times daily.^4^ Only 25% of children aged 6–23 months consume flesh foods and only half consume vitamin A–rich fruits and vegetables in the region.^8^

Improving the diets of infants and young children in Eastern and Southern Africa would contribute to the prevention of malnutrition and is an important component of efforts to achieve the global nutrition targets of the World Health Assembly and Sustainable Development Goals. To make informed recommendations on how to improve diets, insight into specific problematic nutrients, along with foods and feeding practices, is essential. Evidence indicative of the burden of nutrient deficiencies or nutrient intake frequently is available in countries yet are often underused or misinterpreted in decision-making and program design, in part because relevant evidence often comes from disparate sources of varying quality, representativeness, and recency. The absence of guidance on what evidence types should be prioritized to estimate nutrient gaps (ie, shortfalls in the diet that lead to inadequate nutrient intakes) and how to collate and interpret such information, has resulted in limited understanding of the magnitude and significance of nutrient gaps in countries in Eastern and Southern Africa. As a result, specific reference to nutrient gaps is often omitted from policies and programs designed to improve child health and nutrition.

A new methodology called Comprehensive Nutrient Gap Assessment (CONGA) was developed to fill this gap. This approach, detailed in an article by Beal et al^9^ in this *Nutrition Reviews* supplement issue—provides guidance on how to use various types of evidence (eg, biochemical, dietary intake, household consumption) to assess the public health significance of nutrient gaps in a given population. The CONGA was used to assess nutrient gaps during the complementary feeding period in 6 countries in Eastern and Southern Africa: Ethiopia, Mozambique, South Africa, Tanzania, Uganda, and Zambia. After identifying micronutrient gaps, the most micronutrient-dense whole-food sources (ie, those with minimal processing and typically only 1 or 2 ingredients) available in part or all of the countries studied for these micronutrients were identified.

## METHODS

The CONGA methodology used in this analysis is described in detail in the Beal et al article in this supplement.^9^ The population of interest for this CONGA was children aged 6–23 months. Micronutrients assessed were those identified as commonly lacking in the diets of infants and young children during the complementary feeding period: iron, vitamin A, zinc, calcium, iodine, vitamin B_1_ (thiamine), niacin, vitamin B_12_, vitamin B_6_, folate, and vitamin C.^5^

The analysis followed the 8-step method outlined in the CONGA methods paper.^9^ Per step 1, a literature search was conducted for each country to identify information on the 5 evidence types defined as relevant for assessing gaps: 1) biological, clinical, and functional markers; 2) nutrient adequacy of individual diets; 3) nutrient adequacy of household diets; 4) nutrient adequacy of national food supplies; and 5) nutrient-informative food group intake of individuals or households. Other evidence types outside these categories were also considered, including micronutrient supplementation coverage in young children, intake of food groups that are not the only source of but are high in ≥1 nutrients (eg, dairy is high in calcium), and linear programming studies in which problem nutrients were identified (but not prevalence of inadequate intake) in the diets of infants and young children. Evidence was identified by 1) searching relevant global databases, including Demographic and Health Surveys (DHS) and DHS STATcompiler (dhsprogram.com), Multiple Indicator Cluster Surveys (mics.unicef.org), and UNICEF global databases on infant and young child feeding, malnutrition, and iodine (data.unicef.org); and 2) keyword searches in Google and Google Scholar. Keyword search terms were included both for the topic area (eg, nutrient intake in children) as well as survey report types or grey literature (eg, national micronutrient surveys). Once identified, these references were compiled, classified according to evidence type, and sent to the UNICEF Eastern and Southern Africa Regional Office and relevant Global Alliance for Improved Nutrition (GAIN) country offices to identify and collect reports, publications, and data missed in the online search relevant for inclusion in CONGA.

Collated evidence was reviewed to determine if the data met qualifying criteria for inclusion in CONGA. Data based on a sample size <50, representing <10% of the national population, specific to highly vulnerable populations (eg, children living with HIV), and collected >20 years ago were excluded from the analysis. Evidence specific to the age group of interest (children aged 6–23 months) was prioritized; however, evidence for other age groups that overlapped with the target group (eg, children aged 6–59 months), were near to the target group (eg, children aged 5–14 years), or not near but relevant (eg, women of reproductive age [WRA]) were also included where identified. Details on identified qualifying data points were inserted in country-specific spreadsheets. Each spreadsheet summarized the identified data points (including the point estimate, indicator type, and cutoff value if applicable) and their associated metadata (ie, evidence type, geographic representation, recency of data collection, age and sex representation, and sample size). When there was >1 data point for the same specific evidence type (eg, VAD prevalence for children 6–59 months of age and for WRA), the data point most similar to our age group of interest was included and the other estimate was recorded in a comment box to provide additional context rather than inserted as a stand-alone data point.

Two experts (T.B. and J.M.W.) conducted CONGA steps 2–6 to determine gap ratings for all 11 nutrients in each of the 6 countries. In step 2, data points in the country-specific spreadsheets were reviewed and assigned an implied nutrient burden score per the CONGA methodology.^9^ Weights were then assigned to the metadata to calculate an overall evidence weight score for each data point in step 3. Assigning an evidence weight score based on the metadata helped account for the fact that not all data points were equally valuable or robust. Weight scores were systematically applied to the metadata for each data point (see Table 2 in Beal et al^9^), ensuring that the most recent, representative, and relevant data were weighted more heavily when assessing nutrient gaps.

**Table 1:** Number of data points included in CONGA and those that qualified for the quantitative burden score, by country

	Included in CONGA	Qualified for quantitative burden scores
Ethiopia	39	20
Tanzania	22	16
South Africa	21	15
Uganda	31	14
Mozambique	29	15
Zambia	35	13
**Total**	**177**	**93**

**Table 2:** **Number of data points included in CONGA and that qualified for the quantitative burden score, by evidence type and micronutrient** ^a^

Evidence type	Iron	Vit A	Zinc	Ca	Vit B_12_	Folate	Iodine	Vit C	Vit B_1_	Niacin	Vit B_6_	Total
Biological and functional markers	5 (4)	6 (5)	2 (1)	0 (0)	3 (0)	0 (0)	4 (2)	0 (0)	0 (0)	0 (0)	0 (0)	23 (12)
Nutrient adequacy of individual diets	3 (1)	4 (1)	3 (1)	2 (0)	2 (0)	2 (0)	0 (0)	2 (0)	2 (0)	2 (0)	2 (0)	24 (3)
Nutrient adequacy of household diets	0 (0)	0 (0)	0 (0)	0 (0)	0 (0)	0 (0)	0 (0)	0 (0)	0 (0)	0 (0)	0 (0)	0 (0)
Nutrient adequacy of national food supplies	6 (6)	6 (6)	6 (6)	6 (6)	6 (6)	6 (6)	0 (0)	6 (6)	6 (6)	6 (6)	6 (6)	60 (60)
Nutrient-informative food-group intake of individuals or households	6 (6)	6 (6)	0 (0)	0 (0)	0 (0)	0 (0)	6 (6)	0 (0)	0 (0)	0 (0)	0 (0)	18 (18)
Other	5 (0)	9 (0)	7 (0)	7 (0)	7 (0)	1 (0)	0 (0)	1 (0)	7 (0)	7 (0)	1 (0)	52 (0)
Total evidence points	25 (17)	31 (18)	18 (8)	15 (6)	18 (6)	12 (6)	10 (8)	9 (6)	15 (6)	15 (6)	9 (6)	177 (93)

a Data are given as no. with the number of data points that qualified for the quantitative burden scores reported in parentheses. *Abbreviations:* Vit, vitamin, Ca, calcium.

In step 4, the implied burden score and evidence weight scores were used to calculate a quantitatively derived nutrient gap burden rating. The quantitative burden rating was calculated using data only from the aforementioned 5 core evidence types (ie, excluding “other” data) that were collected in the last 10 years on age or sex groups similar to children 6–23 months of age. The data points excluded from this calculation were considered in step 5. A numeric score was calculated for each nutrient in each country by using the weighted mean of the implied burden score (where the evidence weights are the weight scores) and assigned a label of high, moderate, low, or negligible, per CONGA methodology.^9^

In step 5, the quantitative nutrient gap burden scores were reviewed alongside the totality of evidence for each nutrient, including data points that did not meet criteria for inclusion in the quantitative-burden score calculation and additional available information for each data point (eg, temporal trends for data points, where available) to determine whether the final rating assigned to the nutrient gap should deviate at all from the quantitative-derived rating. A final qualitative rating of high, moderate, low, or negligible was assigned to each nutrient for each country, and any deviation from the quantitative burden score was documented and explained.

The certainty of evidence was rated for each nutrient burden (ie, high, moderate, low, or unknown) in step 6 using established CONGA methodology criteria,^9^ which consider the evidence weight scores from step 3 and level of agreement between data points. These criteria-based ratings were also subjected to a final qualitative review, considering all evidence (including data points for which an evidence weight score was not possible) to determine whether the final certainty rating should deviate from the criteria-based rating. Any deviations were discussed and documented.

In step 7, all coauthors, who are subject matter and contextual knowledge experts, reviewed the final qualitative nutrient gap burden and evidence certainty ratings produced in steps 5 and 6, respectively. Disagreements over final qualitative ratings were discussed and critically re-evaluated. Ratings were finalized only when consensus was achieved and documentation of additional considerations or deviations from quantitative burden scores was added. The Supplemental Material contains a spreadsheet with all data points included in each CONGA, data sources, and nutrient gap and certainty scores.

The most micronutrient-dense, regionally available, whole-food sources were determined for identified micronutrient gaps (ie, those that received a gap burden rating and a certainty-of-evidence rating of at least moderate) and potential gaps (ie, those that received a nutrient gap burden rating of at least moderate but a certainty-of-evidence rating of low) using national or US Department of Agriculture^10^ food composition data and consumption patterns from household consumption and expenditure surveys in countries studied.^11^ With the exception of liver, on which data were not always available, foods were eligible for consideration if they were consumed by ≥10% of households nationally (typically over a 1 to 2 week period) in the countries studied. Because many whole foods are good sources of several micronutrients, these foods were also assessed for how well they met the needs of 6 micronutrients critical for child growth and development and likely to be lacking in Eastern and Southern African diets (namely, iron, vitamin A, zinc, folate, vitamin B_12_, and calcium). This metric, called average share of requirements was calculated as the average proportion of daily requirements from complementary foods for these 6 micronutrients on the basis of a 100-g quantity (each micronutrient capped at 100% of daily requirements). The portion size of each food required to achieve an average of 33.3% of requirements (again, capped at 100% of requirements for each micronutrient)—the equivalent of 100% of requirements for 2 micronutrients or 33.3% of requirements for all 6 micronutrients—was also calculated to demonstrate the ideal foods to fill ≥ 2 important micronutrient gaps simultaneously. Adjustments for differences in bioavailability between plant and animal source foods were made for iron and zinc. Additional details on the methodology used to calculate micronutrient density of and average share of requirements for identified foods and determine local availability can be found in other articles in this issue of *Nutrition Reviews*.^11^^,^^12^

### Evidence

A total of 25 evidence sources were identified in CONGA step 1 across the 6 countries (see Supplemental Material for the list of evidence). From these, a total of 177 data points met the aforementioned criteria for inclusion in the CONGA (Table 1). Ethiopia had the most identified data points (*n* = 39) followed by Zambia (*n* = 35), Uganda (*n* = 31), Mozambique (*n* = 29), Tanzania (*n* = 22), and South Africa (*n* = 21). Recency of data collection varied across countries: All eligible data points in Zambia and Mozambique were at least 6 and 7 years old, respectively, whereas Ethiopia, Tanzania, South Africa, and Uganda all had data from 2015 or 2016. All 6 countries had relevant nationally representative data available for several nutrients and evidence types. The variety of data sources differed by country: Qualifying data for Tanzania was almost exclusively from either the 2010 or 2015/16 DHS, whereas Ethiopia had a 2011 National Food Consumption Survey, a 2014/2015 National Micronutrient Survey, and a 2016 DHS survey. Relevance to the age group of interest varied by evidence type and data source; however, the majority of data reviewed were for children younger than 5 years or 6–59 months of age. Slightly more than 50% of all data points collated for CONGA (*n* = 93) met criteria for inclusion in the quantitative-burden score calculation.

Availability of data points for the 5 core evidence types outlined in the CONGA methodology varied. Table 2 lists the number of data points per evidence type by nutrient that 1) met criteria for inclusion in the CONGA, and 2) met criteria for inclusion in the quantitative burden score. Biochemical or functional markers were identified for 6 of the 11 nutrients of interest. The most common biochemical or functional marker available and meeting qualifying criteria was for vitamin A. Data on prevalence of VAD in children younger than 5 years or for children aged 6–59 months were available in all 6 countries—with estimates able to be extrapolated for children age 6–23 months in 4 (either by reported figures or calculation)—and nationally representative in 5 of the countries. The majority of VAD prevalence data was collected before 2015, and the Zambia estimate was collected in 2008 (and thus was excluded from the quantitative-burden score calculation). Estimates of iron deficiency prevalence were available in 5 countries (no estimate in Uganda) but only qualified for inclusion in the quantitative-burden score calculation in four. Three of the iron deficiency prevalence estimates could be extrapolated for aged children 6–23 months. Estimates of anemia prevalence were available for all countries, but these were not used to estimate iron deficiency, because of limited information on the proportion of anemia due to iron deficiency in these populations. Prevalence of zinc deficiency in children under 5 years or 6-59 months of age were available in Ethiopia and Zambia, but the estimate from Zambia was >10 years old. Prevalence data on folate and vitamin B_12_ deficiency were available in Mozambique, Ethiopia, and Zambia, but none of the data points met qualifying criteria for the quantitative-burden rating (estimates were for WRA in Mozambique and Ethiopia and collected in 2008 in Zambia). Estimates of iodine deficiency (either via prevalence of deficiency or median urinary iodine concentration) were available in Mozambique, Ethiopia, Tanzania, and Zambia, but none of the estimates were for children younger than 5 years (estimates were for WRA in 3 of the 4 countries and for children aged 5–14 years in Ethiopia). No biochemical data for any age groups were identified in the literature search for calcium, niacin, thiamin, vitamin C, or vitamin B_6_.

Evidence on the nutrient adequacy of individuals was available only from food consumption surveys in Uganda, Zambia, and Ethiopia; however, only the evidence from Ethiopia qualified for inclusion in the quantitative burden score, because the data from Uganda and Zambia were from 2008. No data points were identified for nutrient adequacy of households in any country for any nutrient.

Estimates on the nutrient adequacy of national food supplies were available for all nutrients in all countries. For several nutrients, this was the only qualifying data point for the quantitative nutrient gap burden score (and, in fact, the only data point for the nutrient identified by CONGA). The quantitative burden scores for niacin, vitamin C, vitamin B_6_, folate, and calcium were determined exclusively by estimates of adequacy of national food supplies. Nutrient-informative food-group estimates for individuals were available for vitamin A and iron in all countries. Nutrient-informative food-group estimates for households were available for iodine in all countries, as well. These data came largely from DHS surveys (eg, consumption of iron- and vitamin A–rich foods in children 6–23 months of age) and other national nutrition surveys. There were multiple data points categorized as “other” for most nutrients.

### Ratings adjustments

During the final review of the quantitative burden score and criteria-based certainty-of-evidence ratings (CONGA step 5), 16 of the 66 nutrient gap burden ratings and 1 of the 66 certainty ratings were adjusted after critical assessment of all evidence available. Of the 16 nutrient gap burden rating changes, the majority (*n* = 11) increased in severity after final review. Ratings changes applied somewhat evenly across many nutrients: Folate, iron, niacin, thiamine, vitamin C, and zinc had 2 changes each, whereas calcium and vitamin C each had 1. The Supplemental Material includes all initial and final nutrient gap burden and certainty of evidence ratings, including justifications for all cases in which the final score deviated from the quantitative burden score or criteria-based certainty score.

Although nutrient gap burden rating adjustments were made for all 6 countries, a total of 6 were made for Uganda and 5 for Ethiopia, compared with 2 for Tanzania and 1 each for Mozambique, South Africa, and Zambia. The ratings adjusted for Mozambique, South Africa, Tanzania, and Zambia were all originally based only on nutrient availability in the national food supply data, and adjustments were made on the basis of consideration of the totality of collated evidence, including “other” data. In Ethiopia, the high number of changes was also a result of the quantitative burden ratings for folate, niacin, vitamin B_12_, and thiamine being based solely on the nutrient availability in the national food supply data. “Other” data points, including biochemical estimates for vitamin B_12_ and folate for WRA, were available and indicated different burden levels. Similarly, the majority of adjustments in Uganda were for quantitative burden ratings based solely on nutrient availability in the national food supply data. The iron ratings in both Ethiopia and Uganda were changed from moderate to high on the basis of all the evidence available and level of agreement between qualifying and nonqualifying data points.

Only 1 certainty rating was adjusted from the criteria-based rating. The criteria-based rating for iron in Uganda was low because all qualifying data points received low overall weight scores. However, the evidence collated, including recent data specific to children aged 6–23 months, was largely in agreement regarding a high burden, increasing our certainty of the nutrient gap burden rating to moderate.

### Final nutrient gap ratings

**Figure 1:**
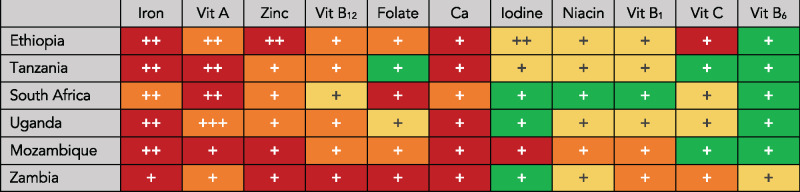
**Gap burden and certainty of evidence for 11 micronutrients among children aged 6–23 months in 6 countries in Eastern and Southern Africa.** Nutrient gap burden is signified by color: red (high burden), orange (moderate burden), yellow (low burden), or green (negligible burden). The number of plus signs (+) represents the certainty of evidence for the nutrient gap burden: 3 (high certainty), 2 (moderate certainty), or 1 (low certainty) evidence. Abbreviations: Vit, vitamin, Ca, calcium

Figure 1 shows the final nutrient gap burden and certainty-of-evidence ratings for the 11 micronutrients studied in all 6 countries in Eastern and Southern Africa. These ratings are not intended to be representative of the entire region; however, the population of children younger than 5 years in these 6 countries represents >50% of the total population younger than 5 years in the region.^13^ Gaps in iron are the highest priority, with an estimated high burden gap in 5 countries and a moderate burden gap in South Africa. Iron deficiency is a primary cause of anemia and can result in cognitive impairment, decreased work productivity, and death.^14^ Gaps for calcium were also estimated as high in 5 countries and as moderate in South Africa (however, as shown in Figure 1, there is lower certainty of evidence for calcium). Calcium deficiency increases risk of development of rickets, but the broader health implications of deficiency in infants and young children are poorly understood.^15^

Other identified nutrient gaps were for vitamin A and zinc, each with three high burden and three moderate burden ratings (however, as with calcium, there was generally lower certainty for zinc). Vitamin A deficiency has severe consequences, even with mild deficiency, including night blindness, increased susceptibility to infections, and death.^16^ And zinc deficiency in children is associated with poor health, increased risk of diarrhea, and impaired cognitive and motor development.^17^^,^^18^ The nutrient gap for vitamin A in South Africa is high in part because of a nationally representative VAD prevalence estimate of 44% in children younger than 5 years (the highest of all 6 countries studied). Both the quantitative-nutrient gap burden score calculation (CONGA step 4) and the qualitative review of the totality of evidence on vitamin A for South Africa (CONGA step 5) considered that this estimate was from 2012 with a low sample size (*n* < 500)^19^ and determined a moderate burden rating was appropriate based on all available evidence.

Other important nutrient gaps across the 6 countries in Eastern and Southern Africa were for vitamin B_12_ and folate. Deficiency of either in infants and young children can have immediate and long-term consequences, including anemia, hindered brain development, and adult depression.^20^^,^^21^ There did not appear to be important gaps in niacin, vitamin B_1_, vitamin B_6_, and vitamin C in all 6 countries. Iodine deficiency was identified as low burden in all countries except Mozambique, where the burden was high. The Mozambique data on iodine, however, were from 2011 and the rating is based on low-certainty evidence.

The majority of nutrient gap burden ratings (*n* = 55 of 66 ratings) had a low certainty of evidence. Only 1 rating—vitamin A in Uganda—was given a high certainty rating. Nutrient gap ratings for iron received a moderate certainty-of-evidence rating in 5 countries. All ratings for vitamin B_12_, folate, calcium, niacin, thiamin, vitamin C, and vitamin B_6_ were based on low-certainty evidence.

### Foods to fill identified and potential nutrient gaps

**Figure 2:**
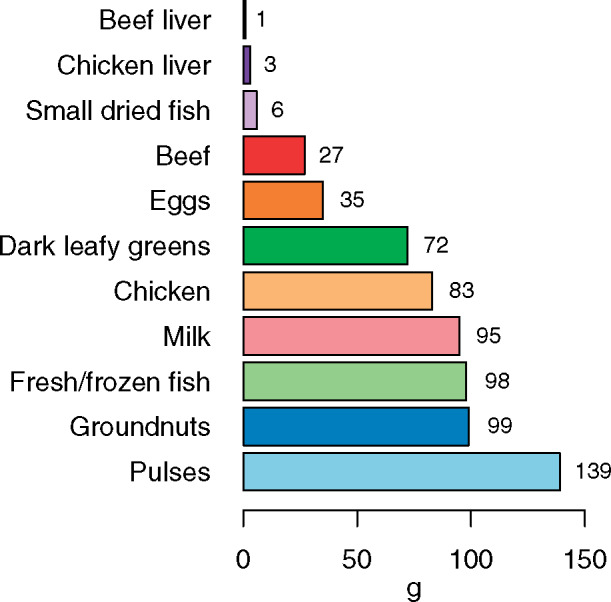
**Portion size needed to achieve an average of 33.3% of requirements for iron, vitamin A, zinc, folate, vitamin B_12_, and calcium from complementary foods in Eastern and Southern Africa (each micronutrient capped at 100% of daily requirements).** The proportion of nutrient requirements from complementary foods was assumed to be 0.98 for iron, 0.87 for zinc, 0.65 for calcium, 0.17 for vitamin A, 0.70 for vitamin B_12_, and 0.60 for folate.^23^ Iron and zinc requirements were adjusted for bioavailability. For iron, we assumed 15% dietary iron bioavailability for animal-source foods and 10% for plant foods; for zinc, we assumed 50% dietary zinc bioavailability for animal-source foods and 30% for legumes, nuts, and seeds^24^

Available whole-food sources rich in the micronutrients with identified and potential gaps are listed in Table 3, including micronutrient densities and average share of requirements. The best whole-food sources of multiple identified or potential micronutrient gaps (ie, iron, zinc, vitamin A, vitamin B_12_, folate, and calcium), in terms of average share of requirements per 100-g portion, are small dried fish, chicken liver, beef liver, eggs, beef, and dark leafy greens. For example, 100 g of small dried fish will achieve an average of 88% of requirements across these 6 micronutrients for children aged 6–23 months. Small dried fish—common in Uganda, Tanzania, Mozambique, and Zambia, but not Ethiopia or South Africa^11^—are also good sources of vitamin D and essential long-chain omega-3 fats, which are important for child development.^25^Figure 2 shows the portion size of each food needed to meet an average of 33.3% of requirements across the same 6 micronutrients. Notably, only 1 g of beef liver, 3 g of chicken liver, 6 g of small dried fish, 27 g of beef, or 35 g of eggs are required to reach this threshold for children aged 6–23 months, demonstrating the importance of these nutrient-dense animal-source foods in young children’s diets. Larger quantities are required, however, for other animal-source foods (eg, chicken, milk, fresh fish) to achieve this threshold. Although a moderate-sized portion (72 g) of dark leafy greens can meet the threshold, a much larger portion of pulses (139 g) would be required to achieve the same outcome. Nutritionally ideal foods may not be available or acceptable in all areas of Eastern and Southern Africa or may only be available seasonally. For differences in common whole-food sources among the 6 countries studied, refer to the Ryckman et al^11^ article in this issue of the journal.

**Table 3:** **Micronutrient densities and average share of requirements per 100 g of foods high in priority nutrients** ^a^

Food	Iron (mg)	Zinc (mg)	Vit A (RAE)	Folate (DFE)	Vit B_12_ (µg)	Calcium (mg)	**Average share of requirements across all 6 micronutrients (%)** ^b^
Small dried fish	10.4	**10.0**	363	19	12.0	**2030**	**88**
Chicken liver	**12.3**	4.0	4139	**569**	19.0	11	84
Beef liver	6.5	5.3	**9442**	253	**70.6**	6	84
Eggs	1.2	1.1	149	44	1.1	50	59
Beef	2.8	6.5	0	8	2.6	8	46
Chicken	1.2	1.9	46	5	0.3	14	42
Dark leafy greens	3.2	0.3	256	42	0.0	98	40
Milk	0.0	0.4	46	5	0.5	113	37
Groundnuts	1.3	2.3	0	86	0.0	57	34
Pulses	2.3	1.1	0	84	0.0	25	29
Mango	0.5	0.1	152	33	0.0	13	26
Carrot	0.3	0.2	852	14	0.0	30	23
Pumpkin	0.6	0.2	288	9	0.0	15	22
Okra	0.3	0.4	14	46	0.0	77	21

a All foods are in the form typically consumed and from a combination of local and US Department of Agriculture food composition^22^ databases, according to Ryckman et al.^11^ Bold numbers indicate highest nutrient density of the specified nutrient or average share of requirements. *Abbreviations:* DFE, dietary folate equivalent; RAE, retinol activity equivalent; Vit, vitamin.

b Average share of requirements across iron, zinc, vitamin A, vitamin B_12_, folate, and calcium per 100 g, assuming requirements from complementary foods for children aged 6–23 months (each micronutrient capped at 100% of daily requirements). The proportion of nutrient requirements from complementary foods were assumed to be 0.98 for iron, 0.87 for zinc, 0.65 for calcium, 0.17 for vitamin A, 0.70 for vitamin B_12_, and 0.60 for folate.^23^ Iron and zinc requirements were adjusted for bioavailability. For iron, we assumed 15% dietary iron bioavailability for animal-source foods and 10% for plant foods; for zinc we assumed 50% dietary zinc bioavailability for animal-source foods and 30% for legumes, nuts, and seeds.^24^

## CONCLUSION

Identification of nutrient and dietary gaps during the complementary feeding period is essential to inform policies and programs designed to improve child health and nutrition.^26^ To identify these gaps, reliable and representative data are required. Anthropometric and aggregate dietary indicators are widely available and often used to monitor and evaluate interventions, guide policies and programs, and track progress related to complementary feeding. Standard global indicators on complementary feeding provide insight into the number of food groups children are consuming and the frequency of meal consumption, and these data are routinely collected for aged children 6–23 months.^26–28^ However, alone, these indicators provide limited insight into the magnitude and significance of nutrient gaps. Using CONGA to assess gaps during the complementary feeding period in a sample of countries in Eastern and Southern Africa allowed us to investigate different evidence types and sources not usually synthesized for assessment of child diets. Only existing evidence was used, with no primary data analysis required. The CONGA methodology also explicitly considers and accounts for data points that disagree on the magnitude of nutrient gaps and for differences in the recency and quality of data points collated. Rating the certainty of evidence for each nutrient gap burden also provides transparency about the quality and breadth of evidence reviewed and allows for advocacy to increase evidence generation for specific nutrients. The findings also enabled investigation into which foods, available in countries studied, were the most nutrient-dense sources of the identified and potential nutrient gaps. The CONGA methodology is standardized so it can be applied across geographic areas and different population groups. A separate series of CONGA for children aged 6–23 months were conducted for all countries in the UNICEF South Asia region, with results summarized by Beal et al in this supplement.^29^

Although CONGA can help expand the evidence base on child diets (and the diets of other populations), there are important limitations to the process. For example, the literature search conducted in CONGA step 1 was not systematic, which may have resulted in omission of qualifying evidence from the review. Much of CONGA is standardized to minimize bias; however, there is purposeful flexibility for reviewers to override quantitative and criteria-based ratings based on qualitative assessment. Thus, the final ratings may reflect the subjective perspective of reviewers. The CONGA methodology addresses this by encouraging multiple reviewers to be engaged in the process and any changes made from quantitative and criteria-based ratings to be discussed, documented, and then validated by subject-matter and local experts.

There was a notable lack of evidence to include in CONGA in the Eastern and Southern Africa region. For all 6 countries, only 25 evidence sources from which to pull relevant data points were identified. The vast majority of nutrient gap burden ratings were based on low-certainty evidence. In contrast, the majority of countries studied in South Asia had either a high or moderate certainty for iron, zinc, vitamin A, folate, and vitamin B_12_ gap ratings, in part due to recent national nutrition or micronutrient surveys available in most countries in the region.^29^ Evidence certainty in Eastern and Southern Africa was strongest for iron and vitamin A, in part because each of these nutrients was most likely to have national biochemical estimates available. Only 2 of the 6 countries assessed in the region—Ethiopia and Mozambique—had national micronutrient surveys conducted within the last 10 years. Similar to the CONGA in South Asia,^29^ there was no biochemical evidence available in the countries studied for vitamin C, niacin, thiamin, or vitamin B_6_ deficiencies. Biochemical and functional marker data should be collected at the national level at least every 10 years (ideally more frequently) to help monitor programs and track progress on child diets. For nutrient adequacy of individuals, there were some data points available from national or subnational food consumption surveys; however, 2 of 3 of these surveys identifed were more than a decade old. In South Asia, no data points for this evidence category were identified in any country in the region.^29^ No data points were identified in Eastern and Southern Africa for nutrient adequacy of households, and only 1 country in South Asia had data points available for this evidence type. The dearth of data from these evidence types across both regions represents a gap in the evidence base to assess nutrient and dietary gaps. New data collection and evidence generation in Eastern and Southern Africa should be prioritized for all 11 nutrients investigated here, but particularly those with a moderate or high burden rating and a low certainty of evidence, such as calcium, zinc, vitamin B_12_, and folate.

Although the identified and potential nutrient gaps varied across countries, all countries included in this analysis had at least a moderate burden in iron, calcium, vitamin A, and zinc, and most had at least a moderate burden in vitamin B_12_ and folate. These gaps are similar to those found in South Asia, where identified and potential nutrient gaps were found for iron, zinc, vitamin A, folate, vitamin B_12_, calcium, and vitamin C.^29^ It should be noted, however, that there may be other nutrient gaps this analysis did not identify because of limited robust evidence for several micronutrients. Increasing the quality of the whole foods consumed by children is an ideal solution to help overcome current nutrient gaps. Animal-source foods were the most nutrient-dense whole-food sources of identified nutrient gaps in Eastern and Southern Africa, particularly small dried fish, liver, eggs, and beef. However, Eastern Africa has the second lowest (behind only South Asia) per-capita supply of meat (29 g/day).^30^ Eastern Africa and Southern Africa both have the second lowest (behind landlocked Central Asia) per-capita supply of fish (16 g/day).^30^ However, food balance-sheet data are likely underestimating fish supplies by not adequately capturing small-scale fisheries, particularly those inland.^31^ The per-capita egg supply in Eastern Africa is exceptionally low (2.5 g/day) and is higher but still in relatively low supply in Southern Africa (16 g/day), mostly because of supply in South Africa.^30^ Social and behavior change communication campaigns and counselling on child diets could encourage the increased allocation of the existing supplies of liver, muscle meat, and small fish to young children. Egg supplies, however, may be too low even for the small quantities required for young children, requiring increased production. Dark leafy greens were also identified as a good source of iron, vitamin A, folate, and calcium. Alternative strategies to fill nutrient gaps include biofortified foods, fortification of staples, fortified complementary foods, point-of-use fortification products such as micronutrient powders and lipid-based nutrient supplements, and periodic micronutrient supplementation. All these strategies are warranted in parts of Eastern and Southern Africa, particularly where food insecurity, social norms, palatability, and desirability make sufficient consumption from accessible, diverse whole foods infeasible. Continued breastfeeding until 2 years of age (or beyond) also makes an important contribution to child diets. Between 12 and 23 months of age, it is estimated that children still receive 35–40% of their energy needs from breastmilk, and it is a good source of essential fatty acids and micronutrients. The nutritional benefit of continued breastfeeding is most evident during child illness, when decreased child appetite may prevent a child from consuming adequate quantities of foods, but breastmilk intake is maintained.^5^

This CONGA analysis helps broaden the understanding of priority and potential nutrient gaps in child diets and contributes to prioritization of strategic actions to improve the quality of children’s diets in these 6 countries as well as the Eastern and Southern Africa region. More research is required to understand the causes of these nutrient gaps, including both supply- and demand-side barriers. The analysis also identified nutrient-dense whole foods to overcome nutrient gaps during the complementary feeding period. More research is needed to understand the primary barriers to consuming these foods, like limited availability, accessibility, affordability, or desirability. Efforts to improve and maintain continued breastfeeding rates in the region should also be prioritized (only 72% of children are still breastfed during the second year of life in the region^32^). Improving the quality of pregnant and lactating women’s diets can also improve their children’s nutrition through improved birth outcomes, nutrient transfers at birth, and more nutrient-dense breast milk.^33^ Behavioral aspects and social and cultural norms influencing feeding practices also need to be addressed. Strategic actions to improve child diets will require engagement and intervention across relevant systems, including food, social protection, health, and water, sanitation, and hygiene. A regional action framework to improve child diets, with priorities across these 4 systems, was recently endorsed by the Southern African Development Community member states, indicating a high level of commitment to push for results at the country level.^34^^,^^35^ In line with the regional action framework, governments in the region need to reorient their food policies and systems to address gaps in the food supply, nutrient availability for identified nutrient gaps, and prioritize improving access and affordability.

## Supplementary Material

nuaa142_Supplementary_Data

## Supporting Information

The following Supporting Information is available through the online version of this article at the publisher’s website

*Supplementary Material:* **List of references that qualified for inclusion in the CONGA in Eastern and Southern Africa**

*Table S1* **Search terms used in the literature review**

*Supplementary Material:* **Spreadsheet of nutrient gap burden and certainty-of-evidence ratings in the 6 countries included in this study**
