# Supplementary material for: Micronutrient gaps during the complementary feeding period in 6 countries in Eastern and Southern Africa: a Comprehensive Nutrient Gap Assessment
Source: Nutr Rev. 2021 Mar 8;79(Suppl 1):16–25. doi: 10.1093/nutrit/nuaa142 (PMC7947982; doi:10.1093/nutrit/nuaa142)
Supplement: nuaa142_Supplementary_Data [file nuaa142_supplementary_data.zip › Supplemental Material - References.docx]

**References that qualified for inclusion in the CONGA in Eastern and Southern Africa**

Baye K, Guyot JP, Icard-Verniere C, et al. Nutrient intakes from complementary foods consumed by young children (aged 12-23 months) from North Wollo, northern Ethiopia: the need for agro-ecologically adapted interventions. *Public Health Nutr.* 2013;16(10):1741-1750.

Beal T, Massiot E, Arsenault JE, et al. Global trends in dietary micronutrient supplies and estimated prevalence of inadequate intakes. *PLoS One.* 2017;12(4):e0175554.

Central Statistical Agency, ICF. *Ethiopia Demographic and Health Survey 2016.* Addis Ababa, Ethiopia: CSA and ICF;2017.

Central Statistical Agency, ICF International. *Ethiopia Demographic and Health Survey 2011.* Addis Ababa, Ethiopia: CSA and ICF International;2012.

Central Statistical Office, Ministry of Health, ICF International. *Zambia Demographic and Health Survey 2013-14.* Rockville, Maryland, USA: CSO, MoH, and ICF International;2015.

Central Statistical Office, Ministry of Health, Tropical Disease Research Centre, et al. *Zambia Demographic and Health Survey 2007.* Calverton, Maryland, USA: CSO and Macro International;2009.

Ethiopian Public Health Institute. *Ethiopian National Food Consumption Survey.* Addis Ababa, Ethiopia: EPHI;2013.

Ethiopian Public Health Institute. *Ethiopian National Micronutrient Survey Report.* Addis Ababa, Ethiopia: EPHI;2016.

Harvey P, Rambeloson Z. *The 2008 Uganda Food Consumption Survey: Determining the Dietary Patterns of Ugandan Women and Children.* 2010.

ICF. The DHS Program STATcompiler. <http://www.statcompiler.com>. Accessed January 10, 2020.

Instituto Nacional de Saúde, ICF. *Inquérito Nacional sobre Indicadores de Malária em Moçambique 2018.* Maputo, Mozambique. Rockville, Maryland, USA: INS and ICF;2019.

Micronutrient Initiative. *The identification of ‘problem nutrients’ and development of Food-based Recommendations for 6-23 month old children in the regions of Tigray, Amhara, Oromia, and SNNPR: Optifood Analysis.* 2015.

Ministerio da Saúde, Helen Keller International, Global Alliance for Improved Nutrition. *Mozambique Micronutrient Survey 2012-13.* Maputo, Mozambique: MISAU, HKI, and GAIN 2017.

Ministerio da Saúde, Instituto Nacional de EstatÌstica, ICF. *Inquérito de Indicadores de Imunização, Malária e HIV/SIDA em Moçambique 2015.* Maputo, Moçambique. Rockville, Maryland, USA: INS, INE, and ICF;2018.

Ministerio da Saúde, Instituto Nacional de EstatÌstica, ICF International. *Moçambique Inquérito Demográfico e de Saúde 2011.* Calverton, Maryland, USA: MISAU, INE, and ICFI;2013.

Ministry of Health, Community Development, Gender, Elderly and Children (Tanzania), Ministry of Health (Zanzibar), et al. *Tanzania Demographic and Health Survey and Malaria Indicator Survey (TDHS-MIS) 2015-2016.* Dar es Salaam, Tanzania: MoHCDGEC, MoH, NBS, OCGS, and ICF;2016.

Ministry of Health, Tropical Disease Research Centre, UNICEF, et al. *Zambia Food Consumption and Micronutrient Status Survey Report.* 2014.

National Bureau of Statistics, ICF Macro. *Micronutrients: Results of the 2010 Tanzania Demographic and Health Survey.* Dar es Salaam, Tanzania: National Bureau of Statistics (NBS) and ICF Macro;2011.

National Bureau of Statistics, ICF Macro. *Tanzania Demographic and Health Survey 2010.* Dar es Salaam, Tanzania: NBS and ICF Macro;2011.

National Department of Health. *South Africa Demographic and Health Survey 2016.* Pretoria: National Department of Health (NDoH);2019.

National Food and Nutrition Commission. *Iodine Deficiency Survey Report: The status of iodine deficiency among pregnant women in Zambia, 2012.* 2012.

Shisana O, Labadarios D, Rehle T, et al. *South African National Health and Nutrition Examination Survey (SANHANES-1).* Cape Town2013.

Uganda Bureau of Statistics, ICF. *Uganda Demographic and Health Survey 2016.* Kampala, Uganda: UBOS and ICF;2018.

Uganda Bureau of Statistics, ICF International. *Uganda Demographic and Health Survey 2011.* Kampala, Uganda: UBOS and ICF International;2012.

World Food Programme. *Trend Analysis: Key Food Security & Nutrition Indicators Mozambique.* 2016.
